# Comparison of Dorsal-to-Ventral Ratios of the Cervical Paraspinal Musculature in French Bulldogs With and Without Cervical Intervertebral Disk Disease and Two Other Breeds Based on CT Scan Measurements

**DOI:** 10.3389/fvets.2021.705632

**Published:** 2021-11-22

**Authors:** Julia Hart, Stefan Rupp, Katinka Hartmann, Carolin Fischer, Pia Düver, Franck Forterre

**Affiliations:** ^1^Division of Small Animal Surgery, Department of Clinical Veterinary Medicine, Vetsuisse Faculty, University of Bern, Bern, Switzerland; ^2^Neurology Department, Tierklinik Hofheim GbR, Hofheim am Taunus, Germany; ^3^VETCARE, Pferde- und Kleintierpraxis AG, Oberwil, Switzerland; ^4^Department of Veterinary Clinical Sciences, Clinic for Small Animals, Justus Liebig University, Giessen, Germany

**Keywords:** French bulldog, IVDD, IVDH, cervical spine, paraspinal musculature, intervertebral disc, angulation

## Abstract

**Objective:** To objectively assess the cervical paraspinal musculature of French bulldogs (FBs) using computed tomography (CT) scan-based measurements, outline differences in other breeds published in the literature, and investigate the potential influence of its cervical paraspinal musculature on predisposed sites for intervertebral disk disease.

**Animals:** Thirty FBs that underwent CT scans of the cervical spine from the skull to C7/T1 were enrolled. Fifteen dogs were patients suffering from intervertebral disk herniation (IVDH group), and 15 dogs underwent CT scans due to brachycephalic obstructive airway syndrome (BOAS group).

**Methods:** At the level of each cervical intervertebral disk from C2/C3 to C7/T1, measurements were performed and statistically analyzed. On the sagittal CT scan reconstruction, the height ratio of the dorsal to ventral paraspinal musculature and the angle of the disk axis to vertebral body length were assessed. On the transverse plane, the area ratio of the dorsal and ventral paraspinal musculature and the ratio of force moments were determined at each intervertebral disk level. Finally, ratios were compared to the values of Labrador retrievers and dachshunds published by Hartmann et al. (1).

**Results:** Comparing the two FB groups, one significant difference was detected in the mean height ratio of the dorsal to ventral paraspinal musculature at the level of C5/C6 (*P* = 0.0092) and C6/C7 (*P* = 0.0076), with IVDH FBs having the more prominent dorsal paraspinal musculature. At the level of C3/C4, a significantly less prominent dorsal paraspinal musculature in FBs than in dachshunds (*P* = 0.0058) and a significantly steeper disk to vertebral body angulation were observed (*P* = 0.0005).

**Conclusion:** Although some incidental differences were found, most parameters did not significantly differ between the BOAS and IVDH FBs. Significant conformational differences in the cervical paraspinal musculature and disk to vertebral body length angulation were found between FBs and two other breeds (chondrodystrophic and non-chondrodystrophic). This study's findings suggest that the paraspinal musculature is an additional biomechanical influencing factor on the preferential sites of IVDH in the cervical spine and that other major factors exist in IVDH development, especially in FBs.

## Introduction

Over the last few decades, French bulldogs (FBs) have gained increased popularity (2). Unfortunately, this breed is more frequently affected by cervical intervertebral disk herniation (IVDH) than other dog breeds (3, 4). FBs suffer from intervertebral disk (IVD) degeneration at an early age (4, 5), thereby inducing IVDH occurring at a median of 4.2 years (3). It is known that FBs are more likely to suffer from cervical IVDH at the level of C3–C4 (3) unlike other chondrodystrophic breeds that are more commonly affected at C2–C3 (6, 7). However, the causative background of this phenomenon remains partially understood (3, 8).

The canine neck and cervical musculature are complex systems due to attachments to different vertebrae, varied lengths of fascicles, and multiple tendinous insertions (9). The cervical spine is considered a chain of solitary vertebrae requiring the support of the surrounding tendons, ligaments, and muscles to control posture during different forms of locomotion. Flexion, extension, lateral bending, axial compression, and torsion have dynamic impacts and biomechanical loads on the spine (10). Additionally, other mechanical influences are transferred into axial compression by the paraspinal musculature and eventually facet joint load, which might influence the load on the intervertebral disk and its degeneration (11, 12).

The forces created by muscles can be estimated by their physiological cross-sectional area (PCSA), fascicle length, and insertion site (13). A previous cadaveric study on mixed-breed dogs evaluated the structural and functional anatomy of canine cervical muscles. It has been revealed that cervical spine muscles have high PCSA values, which are adapted to high force generation (9).

Paraspinal musculature plays an essential role in dynamic stabilization, ensuring locomotion and maintaining the integrity of the cervical spine (14, 15). IVD degeneration might occur due to an imbalance of both static and dynamic spinal stabilizers (15). Although the actual biomechanical situation might be more complex, there is a suspicion that the stronger developed muscle group might create higher forces and exert a stronger moment on the disk (1). In human medicine studies showed that increased spinal muscle activity increases spinal compression and therefore the load on the intervertebral disk (16) and increased muscle activity lead to a prolonged recovery in patients with low-back pain (17).

Cervical paraspinal muscle volume and distribution differ between chondrodystrophic and non-chondrodystrophic breeds, especially regarding the height ratio and vertebral angulation, with the chondrodystrophic breed having the more prominent dorsal portion of the musculature and the steeper vertebral angulation than the non-chondrodystrophic breed in Hartmann et al.'s study (1). While those differences have been observed, the exact influence of this observation in developing IVD degeneration and IVDH remains unknown (1).

For the above-mentioned reasons, it is important to further investigate and fully understand the clinical role of the cervical paraspinal musculature in developing IVD degeneration and IVDH, especially in a predisposed breed, to develop preventive therapeutic optimization and strategies for IVDH patients.

To the author's knowledge, no previous study has addressed the potential influence of the paraspinal cervical musculature conformation on IVD degeneration and consequent IVDH predisposition in FBs. The aims of this study were to investigate the morphometry of the cervical dorsal and ventral paraspinal musculature in FBs and to compare these results with those of a similar study involving dachshunds and Labrador retrievers (1) (1). Another aim was to analyze, within the FB population enrolled in the study, whether differences could be observed in dogs with IVDH compared to dogs without IVDH (2). It is hypothesized that (1) there is a significant difference in the dorsal-to-ventral ratio of cervical paraspinal musculature of FBs compared to values of other breeds, with FBs having a higher height ratio of the paraspinal musculature and a steeper angulation of the cervical spine. Also, it is hypothesized that (2) no significant differences in the dorsal-to-ventral ratio of the cervical paraspinal musculature between FBs with and without IVDH are found, due to the high prevalence of IVDH in this breed.

## Materials and Methods

### Patients

Computed tomography (CT) scans of FBs performed between January and December 2020 in a referral veterinary hospital for reasons unrelated to the study were retrospectively evaluated. Quantitative analysis was performed using the same protocol described by Hartmann et al. (1). Dogs included in this retrospective study were divided into two groups. The first group included FB patients with CT-confirmed acute cervical IVDH, and the second group included FB patients who underwent CT because of brachycephalic obstructive airway syndrome (BOAS) without clinical signs indicative of cervical disk herniation (cervical pain, neurological deficits). Exclusion criteria were incorrect positioning, poor image resolution, or cervical anomalies, such as cervical spondylomyelopathy or severe vertebral anomalies that could have influenced measurements of the paraspinal musculature.

### CT Examination

All dogs underwent general anesthesia and received the same anesthesia protocol during the whole procedure. The patients received a peripheral intravenous catheter in one of the saphena lateralis veins. Anesthesia was induced with diazepam (Ziapam, 5 mg/ml, Ecuphar, Belgium) 0.5 mg/kg IV and propofol (Narcofol, 10 mg/ml, cp-pharma, Germany) IV titrated to effect. After endotracheal intubation of the animal, anesthesia was maintained with isoflurane (Isofluran CP, 1 ml/ml, cp-pharma, Germany) 1–3 Vol% and oxygen. All dogs were positioned in standardized ventral recumbency, with their front limbs extended caudally to achieve optimal visualization of the entire cervical spine. Hartmann et al. positioned dogs in dorsal recumbency, with their legs extended cranially (1). CT scans were performed from the head to T2 using a 16-slice helical CT scanner (Aquilion®, Canon medical systems GmbH, Germany).

### Image Analysis and Measurements

**Figure 1:**
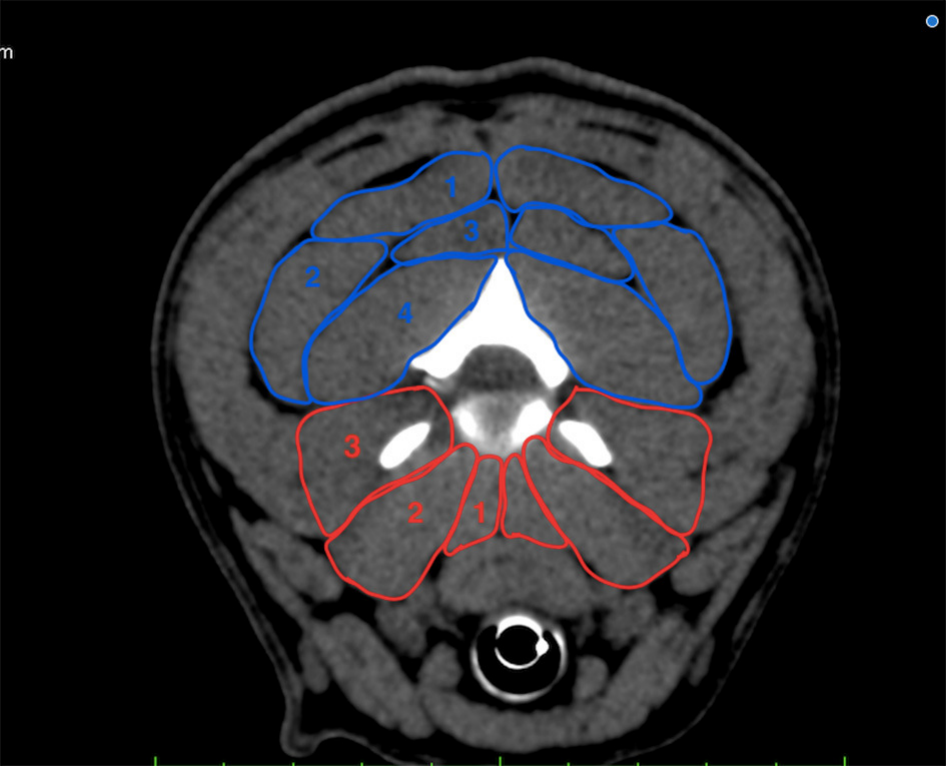
Transverse plane depiction of the paraspinal muscles included in the measurement at the level of C2/C3. Dorsal group (blue): M. biventer cervicis (1), M. longissimus atlantis et capitis (2), M. complexus (3), M. obliquus capitis caudalis (4); ventral group (red): Mm. intertransversarii (3), M. longus capitis (2), M. longus colli (1).

Measurements were performed on sagittal reconstructions and transversal images from C2/C3 to C7/T1. The measurement method was identical to the method described by Hartmann et al. A height ratio and angle measurements in the sagittal plane and an area ratio, alongside a ratio of moments in the transversal plane, were measured (1). All measurements were performed in the vertical axis of each IVD space to estimate the possible impact of the paraspinal musculature on IVD degeneration and IVDH development (1). CT images were analyzed using the Horos Dicom Image Viewer (Horos DICOM Image Viewer Version 3.3.6, Horos™, USA). Muscles measured in the CT scan, depending on the IVD level from cranial to caudal, were identical to those of the previously mentioned study and included M. obliquus capitis caudalis, M. complexus, M. longissimus atlantis, M. longissimus capitis, M. biventer cervicis, M. spinalis cervicis, Mm. multifidi in the dorsal paraspinal musculature group, M. longus colli, M. longus capitis, M. omotransversarius, M. scalenus medius, and Mm. intertransversarii in the ventral paraspinal musculature group (1, 18, 19) (Figure 1). The measurements are briefly explained below; however, for detailed information, Hartmann et al.'s study (1) was referenced. Results of the measurements of the BOAS group were compared to the ratios in dachshunds and Labrador retrievers (1) to determine significant differences between the three breeds.

### Sagittal Plane

#### Height Ratio

The height ratio was determined to show the strength and thickness of the dorsal and ventral portions of the paraspinal musculature. Two anatomical distances were evaluated at the level of each cervical IVD space: distance v (dorsal tracheal margin to ventral margin of the IVD) and distance d (ventral arcus vertebrae margin to dorsal musculature-fat transition).

**Figure 2:**
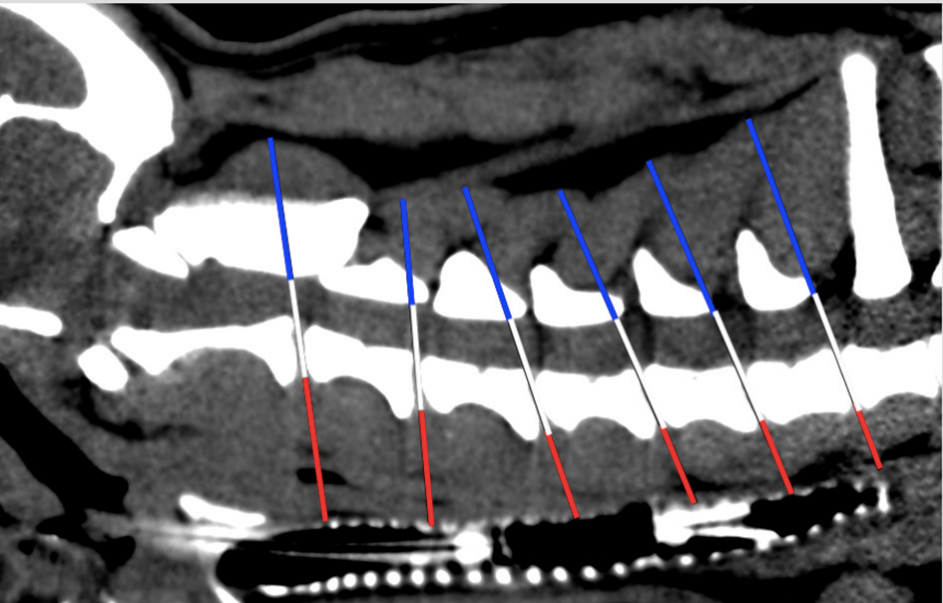
Scheme of height ratio measurement in sagittal plane CT image. White line: level of each intervertebral disk space of the cervical spine; red line: distance v; blue line: distance d.

Distance d was divided by distance v to evaluate the height ratio of the paraspinal musculature (1) (Figure 2). A height ratio > 1 shows that muscle mass is greater in the dorsal paraspinal musculature. For each cervical level, mean distances were calculated in both the BOAS and IVDH groups to determine whether significant differences existed between the two groups (1).

#### Angle Measurement

**Figure 3:**
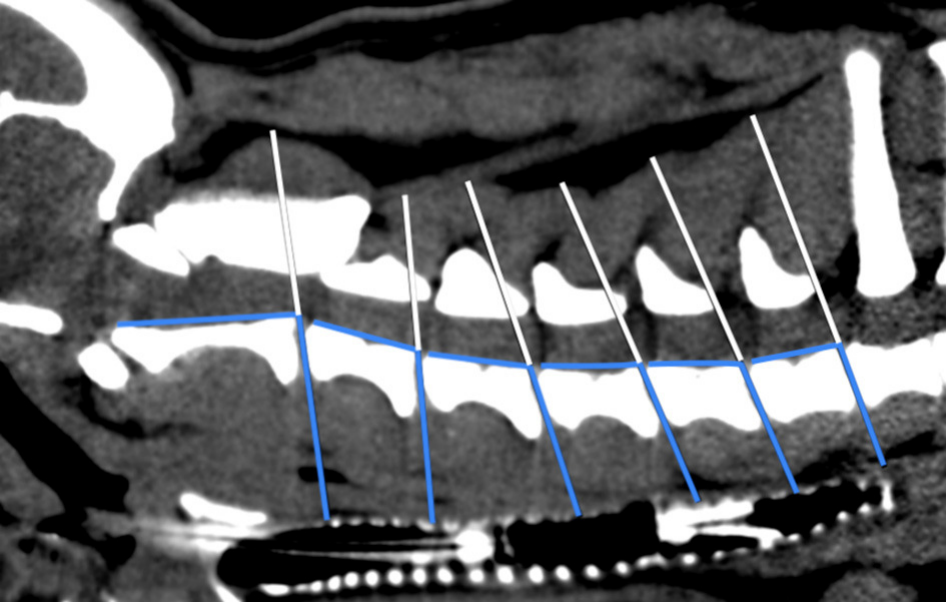
Scheme of angle measurement in sagittal plane CT image. White line: level of each cervical intervertebral disk space; blue lines: angles between IVD spaces in relation to the axis of the cervical vertebral column.

Angles between the IVD spaces regarding the axis of the cervical vertebral column were measured (1). Angle measurement aimed to evaluate disk angulation and its potential influence on the load on each cervical IVD. Consequently, a line was drawn on the floor of the vertebral canal connecting the cranial and caudal endplates of the cervical vertebral bodies. After that, a second line was drawn from every caudal endplate in the same axis as the IVD space to the dorsal margin of the trachea. The angle was finally determined between those two lines at each cervical IVD space (Figure 3). For both FB groups, a mean angle was calculated and compared.

### Transverse Plane

#### Area Ratio

**Figure 4:**
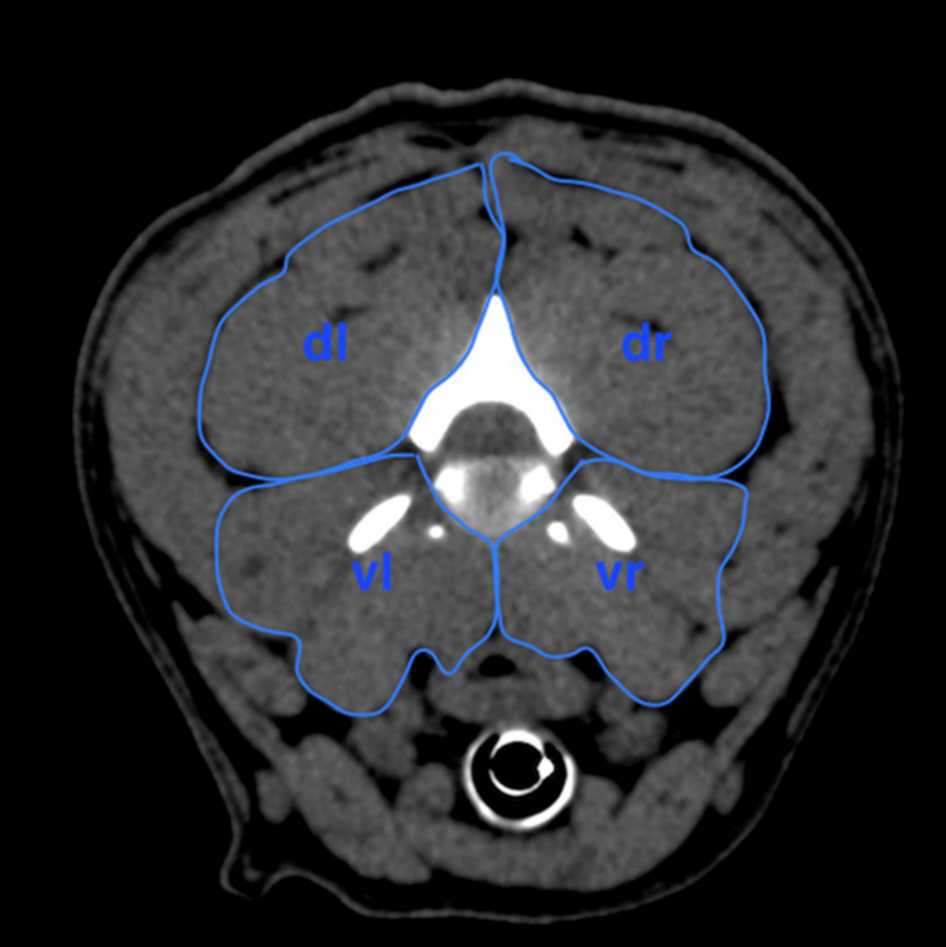
Scheme of measurement of the areas in the four quadrants of the cervical paraspinal musculature at the intervertebral disk level of C2/C3 in transversal plane to calculate area ratio. dr, dorsal right area; dl, dorsal left area; vr, ventral right area; vl, ventral left area.

The areas of the cervical paraspinal muscle groups were outlined on the CT image at each IVD space of the cervical spine (Figure 4) to show the muscle mass of the dorsal and ventral parts of the transverse plane. The computer program was used to calculate the values of these areas. Muscle groups of the paraspinal musculature were divided into dorsal and ventral, left and right areas leading to a ventral right (vr) and a ventral left (vl) muscle area, which were added up to the ventral paraspinal musculature (vrl). Dorsal right (dr) and dorsal left (dl) muscle areas were added up to the dorsal paraspinal musculature (drl). Finally, the area ratio for each cervical segment was calculated by dividing drl by vrl (1). Mean area ratios of both the IVDH and BOAS groups were determined to evaluate statistically significant differences.

#### Ratio of Moments

**Figure 5:**
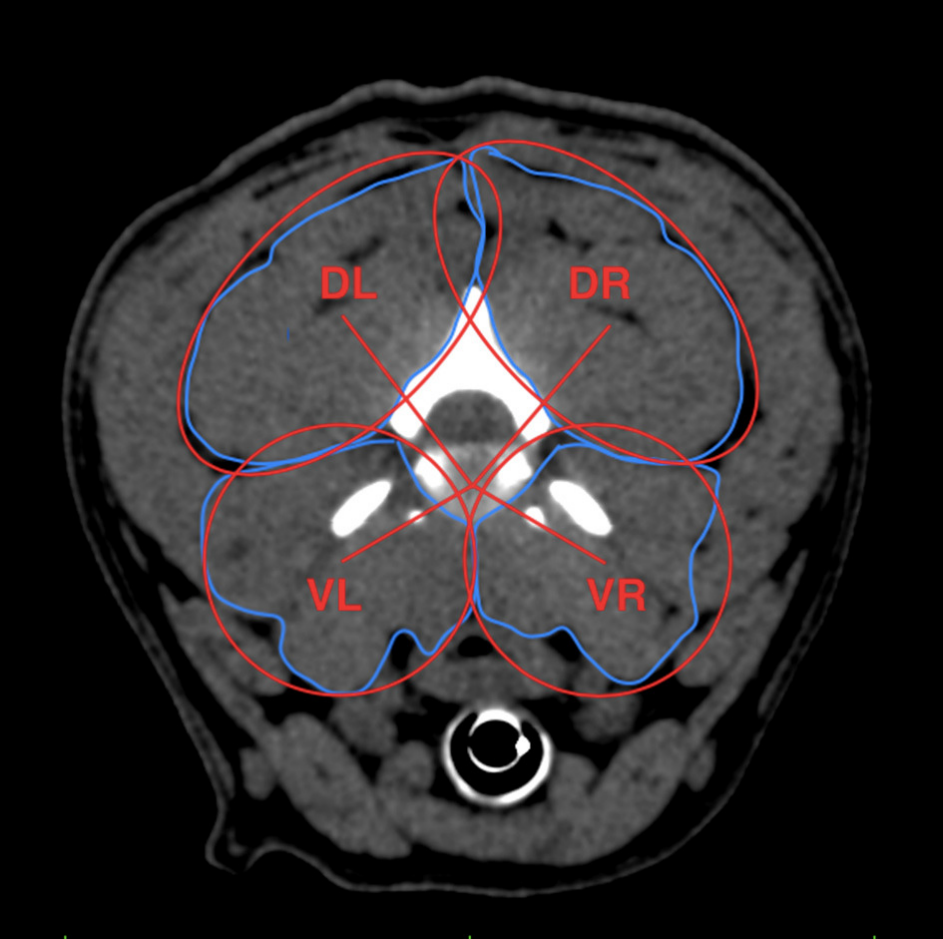
Measurement scheme describing the distances between the disk center and assumptive center of the muscle groups calculated with ellipsoid models to further determine respective relation of moments. Red lines describing lever arms of each paraspinal muscle group. DR, dorsal right; DL, dorsal left; VR, ventral right; VL, ventral left.

The ratio of moments was calculated to determine the forces affecting the vertebral body and to compare the dorsal and ventral load. Four ellipses were placed over the vr, vl, dr, and dl muscle groups to measure the area ratio. To calculate the ratio of moments, the center of the assumptive muscle group was identified with the ellipsoid models. The distances from the muscle group centers to the disk center at each level of the cervical spinal cord were measured, describing the lever arms of each of the four muscle groups (VR = ventral right, VL = ventral left, DR = dorsal right, DL = dorsal left) (Figure 5). Finally, the relation between the four moments was defined by the equation described by Hartmann et al. (1). Mean ratio of moments was calculated for both FB groups to determine statistically significant differences.

### Influence of Cervical Paraspinal Musculature on the IVDH Site

It was evaluated whether the cervical paraspinal musculature influenced the IVDH site in the FB group with IVDH. It was determined at which intervertebral disk space the IVDH was located in the dogs of this group. Following this, it was evaluated whether there were significant differences in the cervical paraspinal musculature between the two FB groups at this IVD space.

### Statistical Analysis

Statistical analysis was performed using Microsoft Excel 2019 (Microsoft Corporation, Version 16.52, USA, 2021) and GraphPad Prism 9 (GraphPad Software, Version 9.2.0, USA, 2021).

A two-sided unpaired *t*-test was performed to evaluate differences between the IVDH and BOAS groups and between FB (BOAS group) and dachshunds and Labrador retrievers (1) separately to outline statistically significant differences between the breeds. BOAS FBs were used for statistical comparison to other breeds, including dachshunds and Labrador retrievers.

Histograms confirmed that the data followed a normal distribution. After confirmation, a *t*-test was performed at each IVD space separately. *P*-values < 0.05 were considered statistically significant and were unadjusted. The Bonferroni correction was applied to correct the level of significance for multiple comparisons.

A power analysis (power = 0.75) showed a sufficient number of samples in each group (*n* > 14) to outline statistically significant differences (α = 0.05).

## Results

CT scans of the cervical spine of 30 FBs were analyzed, and the paraspinal musculature of the cervical spine was evaluated using the measurements and ratios mentioned above. Fifteen FBs with IVDH and 15 without clinical signs of cervical IVDH but suffering from BOAS were included. IVDH patients had a median age of 5 years (range 2–12 years), with a median body weight of 13.0 kg. BOAS patients had a median age of 3 years (range 1–8 years) and a median body weight of 13.2 kg. There was no difference in sex distribution between the two groups (Table 1).

**Table 1:** Demographics of the patients in both IVDH-group and BOAS- group, including gender (f, female; m, male; s, spayed; c, castrated), age (years), body weight (kg), and site of IVDH.

**IVDH-group**				**BOAS-group**		
**Gender**	**Age (years)**	**Body weight (kg)**	**Site of IVDH**	**Gender**	**Age (years)**	**Body weight (kg)**
m	6	14.5	C5/C6	f/s	3	10.8
f/s	6	12.6	C2/C3	f	1	15.1
f	4	8.7	C4/C5	f/s	2	13.4
m/c	6	18.9	C4/C5	f	2	8.0
f/s	4	8.8	C2/C3	f/s	3	11.0
m/c	3	14.0	C4/C5	f/s	7	10.9
f/s	5	13.0	C4/C5	m/c	4	13.9
f/s	6	11.8	C4/C5	m/c	4	16.2
f/s	3	8.0	C3/C4	f	4	11.3
f/s	9	14.6	C3/C4	f	2	10.5
m/c	5	12.5	C3/C4	f	1	10.5
f/s	12	12.4	C3/C4	m	5	15.4
m	5	14.5	C3/C4	m/c	3	17.0
m	4	16.5	C6/C7	m	2	14.6
m/c	2	13.8	C3/C4	f/s	8	13.2

### Sagittal Plane Measurement

#### Height Ratio

**Figure 6:**
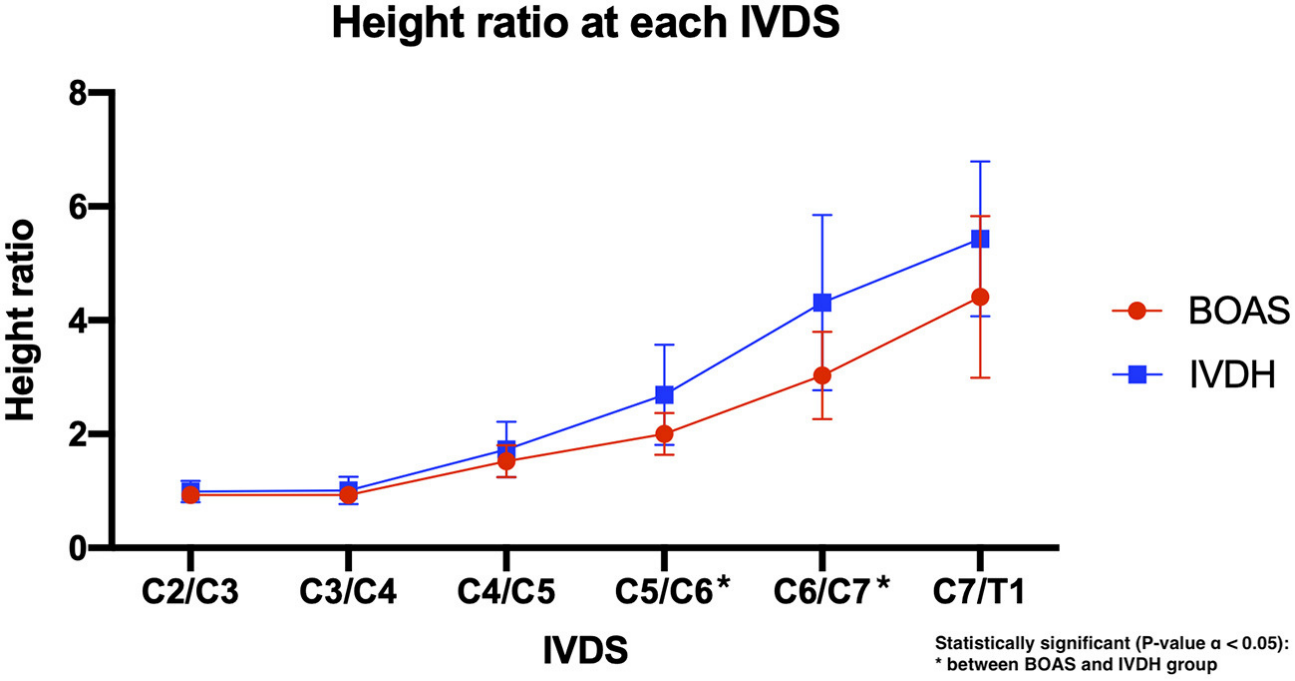
Line graph showing the mean dorsal-to-ventral height ratio of the cervical paraspinal musculature in French bulldogs of the BOAS group (red line) and the IVDH group (blue line).

In both groups, the height of the dorsal paraspinal musculature increased throughout the cervical spine from cranial to caudal (C2/C3–C7/T1). A significant difference, with a higher mean height ratio of the cervical paraspinal musculature, was detected in the IVDH FBs compared to the BOAS FBs in the caudal cervical spine at the levels of C5/C6 (*P* = 0.0092) and C6/C7 (*P* = 0.0076) (Table 2; Figure 6).

**Table 2:** Comparison of height ratio, angles, area ratio, and ratio of moments of the different intervertebral disk spaces from C2/C3 to C7/T1 in French bulldogs of the IVDH and BOAS group.

	**IVDS**	**Height ratio**			**Angles**			**Area ratio**			**Ratio of moments**		
		**Mean**	**(SD)**	***t*-test *P*-value**	**Mean**	**(SD)**	***t*-test *P*-value**	**Mean**	**(SD)**	***t*-test *P*-value**	**Mean**	**(SD)**	***t*-test *P*-value**
BOAS	C2/C3	0.93	0.12	0.3100	103.31	5.50	0.3932	1.39	0.20	0.1713	1.93	0.38	0.1027
IVDH		0.99	0.19		101.65	4.97		1.49	0.19		2.18	0.43	
BOAS	C3/C4	0.93	0.15	0.2829	113.55	4.06	0.6900	1.59	0.22	0.1839	2.81	0.54	0.1857
IVDH		1.01	0.24		114.05	2.57		1.49	0.18		3.08	0.55	
BOAS	C4/C5	1.52	0.28	0.1606	116.29	4.32	0.5293	1.54	0.20	0.4067	3.02	0.55	0.3219
IVDH		1.73	0.49		117.34	4.70		1.48	0.19		3.23	0.59	
BOAS	C5/C6	2.00	0.37	0.0092	117.20	5.76	0.5522	1.48	0.27	0.0734	3.09	0.89	0.2461
IVDH		2.69	0.88		118.38	4.95		1.66	0.26		3.45	0.77	
BOAS	C6/C7	3.03	0.77	0.0076	114.49	4.88	0.0542	2.32	0.33	0.3925	5.91	1.35	0.1221
IVDH		4.31	1.54		118.09	4.93		2.42	0.30		6.69	1.33	
BOAS	C7/T1	4.41	1.42	0.0542	107.84	6.82	0.3181	3.34	0.67	0.1882	10.45	2.92	0.1375
IVDH		5.43	1.36		109.91	3.96		3.67	0.67		11.96	2.47	

*Numbers with a significant difference (P < 0.05) between these two groups are marked in red*.

#### Angle Measurements

**Figure 7:**
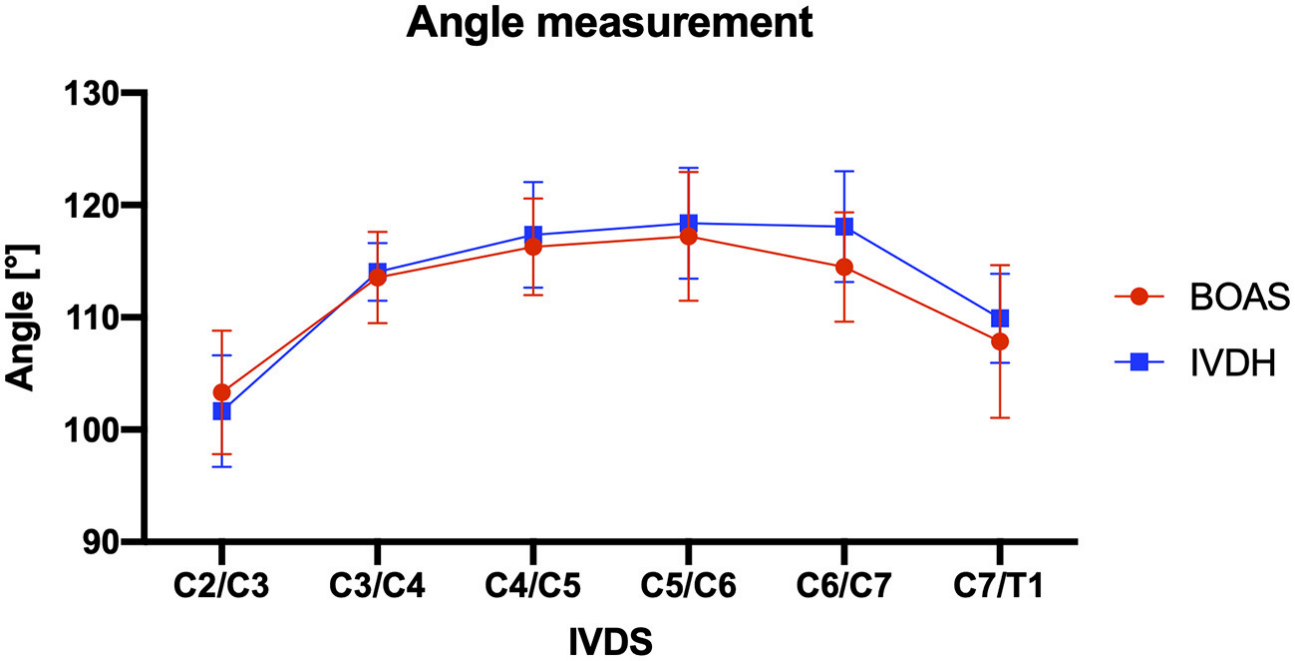
Line graph showing mean angles of intervertebral disk to vertebral body at each IVDS of the cervical spine from C2/C3 to C7/T1 in French bulldogs of the BOAS group (red line) and the IVDH group (blue line).

There was no statistical significance found in mean IVD angulation to the vertebral body length axis in FBs with and without IVDH (Table 2). At the intervertebral disk space of C6/C7, the highest divergence in the angulation of the IVD to the vertebral body was determined but was not statistically significant (Table 2; Figure 7).

### Transverse Plane Measurements

#### Area Ratio

**Figure 8:**
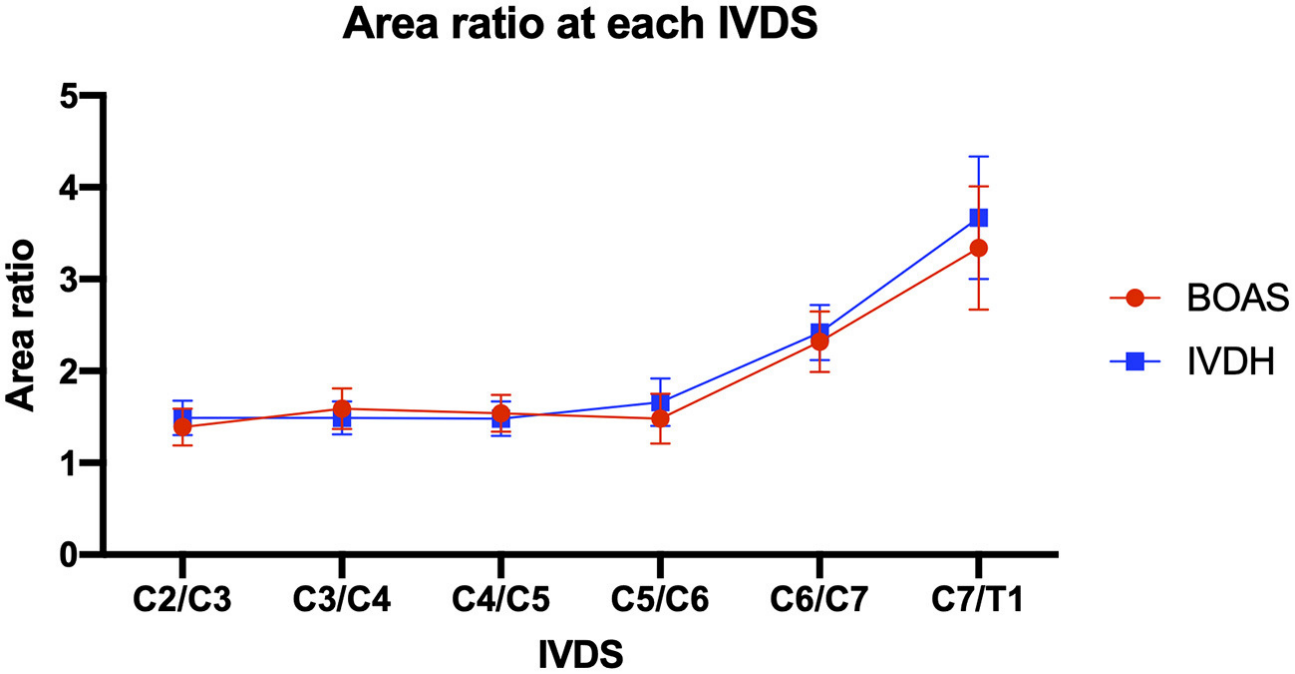
Line graph showing dorsal-to-ventral mean area ratio of cervical paraspinal musculature in IVDS from C2/C3 to C7/T1 in French bulldogs of the BOAS group (red line) and the IVDH group (blue line).

The mean area ratio of the dorsal-to-ventral cervical paraspinal musculature showed consistent values from the IVD space of C2/C3 to C5/C6. It increased rapidly in the IVD space of C6/C7 and C7/T1 in both groups (Figure 8), but there was no statistically significant difference detectable in dorsal-to-ventral mean area ratios between the patients of these two groups (Table 2).

#### Ratio of Moments

**Figure 9:**
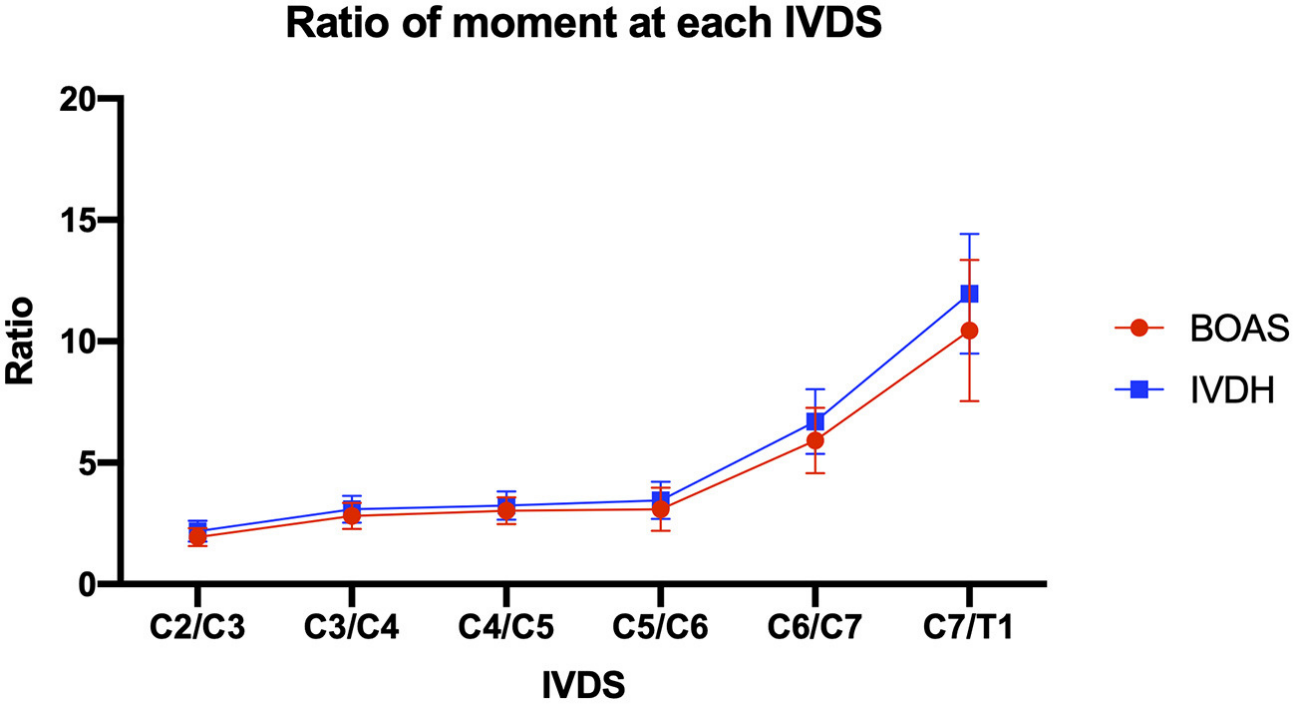
Line graph showing mean ratio of moments of the cervical paraspinal musculature from IVDS of C2/C3 to C7/T1 in French bulldogs of the BOAS group (red line) and the IVDH group (blue line).

No statistically significant difference was detected between IVDH and BOAS FBs (Table 2; Figure 9).

### Influence of Cervical Paraspinal Musculature on the IVDH Site

The sites of cervical IVDH of dogs in the IVDH group were C2/C3 (2/15), C3/C4 (6/15), C4/C5 (5/15), C5/C6 (1/15), and C6/C7 (1/15) (Table 1). The primary localization was C3/C4 and C4/C5. No significant differences in the paraspinal musculature between the two FB groups were detected on these levels.

### Comparison to Labrador Retriever and Dachshund

Statistically significant differences were detected in height ratio, angle measurements, area ratio, and ratio of moments of the paraspinal musculature along the cervical IVD spaces from C2/C3 to C7/T1 between BOAS FB, Labrador retrievers and dachshunds.

**Figure 10:**
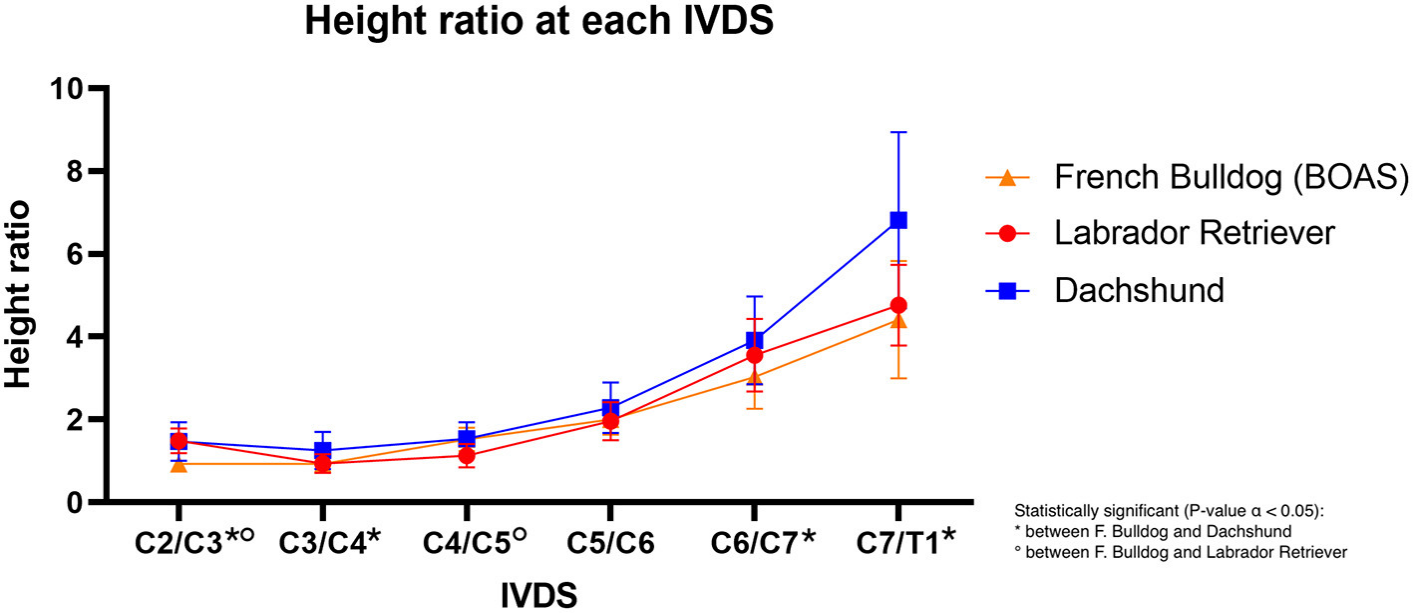
Line graph showing the mean dorsal-to-ventral height ratio of the cervical paraspinal musculature in French bulldogs (orange line), Labrador retriever (red line), and dachshund (blue line).

Focusing on the height ratio (Supplementary Table 3), one of the main findings was that the dorsal paraspinal musculature was statistically significantly less prominent at the level of C3/C4 in FBs (=0.93) compared to dachshunds [=1.25 (15)] (*P* = 0.0143). No statistically significant difference was found compared to Labrador retrievers at this spine level. Also, at the level of C2/C3, FBs (=0.93) had a significantly less prominent paraspinal musculature than dachshunds [=1.47 (15)] (*P* = 0.0002). At this cervical spine level, there was also a significant difference between FBs (=0.93) and Labrador retrievers [=1.49 (15)], with FBs having the less prominent dorsal paraspinal musculature (*P* = 0.0001) (Figure 10; Supplementary Table 3).

**Figure 11:**
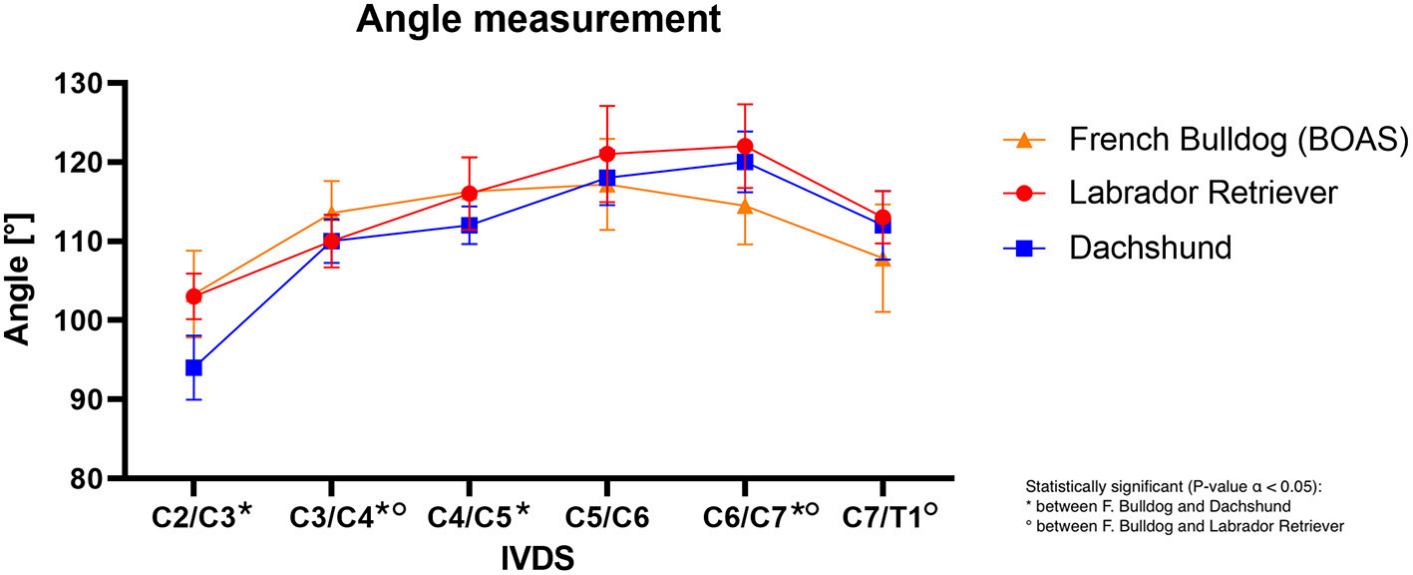
Line graph showing mean angles of intervertebral disk to vertebral body at each IVDS of the cervical spine from C2/C3 to C7/T1 in French bulldogs (orange line), Labrador retriever (red line), and dachshund (blue line).

Concerning the angulation of the intervertebral disk space to the length of the vertebral body, there was steeper angulation in FBs in the cranial cervical spine compared to the other breeds. However, this constellation changed in the caudal cervical spine, with Labrador retrievers and dachshunds having an overall steeper angulation than FBs. Especially at the levels of C2/C3 and C3/C4, FBs (C2/C3 = 103.3°; C3/C4 = 113.5°) had a significantly steeper angulation than dachshunds (C2/C3 = 94°; C3/C4 = 110°) (C2/C3 *P* = 0.0001; C3/C4 *P* = 0.0093) (Supplementary Table 3) (1). In the caudal cervical spine, FBs differed significantly from Labrador retriever, with the Labrador retriever having steeper angulation (1) (Figure 11; Supplementary Table 3).

The area ratio and ratio of moments of the paraspinal musculature in the FB cranial cervical spine exceeded those of Labrador retrievers and dachshunds. This observation was not valid in the caudal cervical spine, where the dorsal musculature in Labrador retrievers and dachshunds became more prominent (1) than in French bulldogs (Supplementary Table 3).

## Discussion

The first hypothesis of this study mentioned above could not be confirmed because two significant differences in the dorsal-to-ventral ratio of the cervical paraspinal musculature between the two FB groups with IVDH or BOAS were detected. Confirming the second hypothesis, several significant differences between the dorsal-to-ventral ratios of FB compared to the two other breeds (dachshund and Labrador retriever), investigated in another study, were found (1). Another interesting finding was that the cervical paraspinal musculature did not influence the IVDH site in the cervical spine.

Addressing the demographics of this study, the median age of BOAS FBs was 3 years, whereas the median age of FBs that presented to the veterinary hospital with cervical IVDH was 5 years. This finding supports the outcome of other studies, which revealed cervical and thoracolumbar IVD degeneration, and IVDD typically develops at the age of 3–7 years in chondrodystrophic dogs (4, 5, 20).

In this study, two significant differences between the two groups of FBs were found. There was a higher mean height ratio of the cervical paraspinal musculature detected in the IVDH FB group than in the BOAS group in the caudal cervical spine at the levels of C5/C6 (*P* = 0.0092) and C6/C7 (*P* = 0.0076). A similar trend was detected at the level of C7/T1 but was not statistically significant. This is possibly because the paraspinal musculature might have a larger anatomical and biomechanical influence on IVD degeneration development and following IVDH than expected. However, the complete biomechanical influence of the paraspinal musculature on IVDH development and its clinical impact remains unclear.

Another aim was to detect the potential influence of the paraspinal musculature on the cervical IVDH site in FBs. The primary localizations of IVDH in the cervical spine in our study were C3/C4 and C4/C5. No significant differences in the paraspinal musculature between the two FB groups were detected on these levels. This probably indicates that the influence of the paraspinal musculature should not be projected onto one specific IVDH site and much more as a biomechanical factor for the whole cervical spine. This contrasts with the findings mentioned above and could still be due to the relatively small sample size that probably remains insufficient to answer this question completely.

The authors compared the results of this study to a prior report with an identical methodology that addressed the subject of paraspinal musculature as a potential influencing factor on IVD disease (IVDD) development (1). In this study, Hartmann et al. compared the cervical paraspinal musculature of dachshunds as a representant of a chondrodystrophic breed and Labrador retrievers as a non-chondrodystrophic breed. Some significant differences in height and area ratios, along with the ratio of moments and differences in disk angulation between those two breeds, especially in the upper cervical spine, were observed (1). Those findings might support the preferential occurrence of IVDD in the upper cervical spine of chondrodystrophic dogs (1).

It was remarkable when the values of Labrador retriever and dachshund were compared to the values of the FBs that there were significant differences in height and area ratio, the ratio of moments, and the angulation from the IVD space to the length of the vertebral body along the cervical spinal cord (1). One of the most noticeable differences was that the dorsal paraspinal musculature of FBs was less prominent than in dachshunds, especially at the level of C3/C4, which is the most common site of cervical FB IVDH (3). Also, at the level of C2/C3, which is the third most common FB IVDH site (3), the dorsal paraspinal musculature was significantly more prominent in dachshunds than in FBs. This result induces the assumption that the muscle strength of the dorsal paraspinal musculature might have a biomechanical impact in preventing IVDH because of the paraspinal musculature stabilizing the cervical spine. These findings were supported by studies from human medicine which showed that increased spinal muscle activity increased spinal compression and therefore the load on the intervertebral disk (16) and that increased muscle activity lead to a prolonged recovery in patients with low-back pain (17).

Focusing on the angulation of the IVD space to the vertebral body length, it was peculiar that the maximum angle of FBs was located at the levels of C4/C5 and C5/C6 compared to the maximum angulation at C6/C7 in dachshunds and Labrador retrievers. Particularly in the cranial part of the cervical spine, and especially at the level of C3/C4, FBs had a significantly steeper angulation compared to dachshunds and Labrador retrievers (1). This supports the hypothesis that steeper IVD confirmation might be another additional biomechanical risk factor for developing IVDH due to a significantly different compressive loadbearing and force transmission (3). It was conceivable that synergizing both findings, less steep angulation and a lesser amount of paraspinal dorsal musculature, might provide a further biomechanical possible explanation for cervical IVDH occurring preferentially at C3/C4 in FBs.

Referring to the results of height ratio, area ratio, and relation of moments, the dorsal paraspinal musculature was generally more prominent than the ventral paraspinal musculature group. However, Hartmann et al. detected a statistically significant difference in the ratio of moments between Labrador retriever and dachshund at the level of C2/C3, with dachshund having the lower ratio (1). The smaller ratio was indicative of a more prominent ventral paraspinal musculature at this level, which might indicate a higher strength. It could play a biomechanical role because of a higher tensile strain on the dorsal anulus fibrosus, which is pathologically less resistant (5, 21), and could induce disk herniation for IVD degeneration (1). It was hypothesized that this finding might play a role in developing IVDD in dachshunds at C2/C3, which is the most commonly affected site for cervical IVDH in this breed. However, our study could not identify this finding when focusing on the predisposed cervical sites of C3/C4, C4/C5, and even C2/C3 (3) in FBs. Also, in the study of Hartmann et al., this difference was not seen at the level of C3/C4 in dachshunds, which is also a frequently affected site (22, 23). This showed that the biomechanical role of the paraspinal musculature and its influence on developing IVD degeneration and secondary IVDH remains partially understood. Furthermore, other anatomical and biomechanical conditions, especially in chondrodystrophic breeds like dachshund and FB, which might interact during the development process of IVDD and IVDH, should not be neglected. These circumstances include malformations of the vertebral column and vertebra itself (24, 25), complete degeneration of the disk at a very young age, and a more dorsally located nucleus pulposus (5, 26, 27).

Several limitations of this study must be considered. First, the retrospective character of the study should be reflected. Consequently, it was not possible to create age- or body weight-matched groups. Due to the relatively uniform young age of the patients enrolled, it was presumed that age itself might not significantly influence the shape or strength of the paraspinal musculature in our study. The median body weight was almost the same for both groups of FBs. Evaluating Body Condition Score (BCS) would have been more accurate but was unfortunately unavailable due to the retrospective character of the study. Furthermore, to exclude body weight as an influencing factor, ratios were used to describe the dorsal and ventral paraspinal musculature proportions. Another limitation was the fact that the median age in the BOAS group was 3 years. Patients suffering from this disease were hospitalized relatively early for diagnostic work-up and surgery. Hence, it could not be anticipated that these patients would not develop IVDH during their lifetime. Furthermore, the probability of developing IVDH at a median age of 4 years (3) should be considered.

Another possible major limitation was the caudally extended legs compared to the study by Hartmann et al., where the legs were extended cranially (1). This difference was due to the retrospective characteristics of both studies. It was anticipated that the shape of the cervical paraspinal musculature, with its deep position directly beside the bony cervical spine, was not affected by the limb position. Another reason for the caudally extended legs was the possibility of taking a smaller Field of View, inducing fewer hardening artifacts and photon starvation. Furthermore, all CT scans were performed under general anesthesia. Muscle relaxation induced by anesthesia might have influenced the measured values. Since ratios were considered, this error might have been minimized.

Finally, this study focused only on one single possible etiological aspect in developing IVDD and subsequent IVDH. Since IVDD has a complex, multifactorial etiology (28), other influencing factors of developing IVDH in chondrodystrophic dogs, such as FBs, should always be considered when discussing potential risk factors.

Further studies, including a larger sample size, might be needed to understand this phenomenon entirely because dachshund is also predisposed to developing IVDH. It might also be helpful to include healthier dogs in future investigations on this topic or to compare two groups of dachshund and Labrador retriever with and without IVDH to understand the biomechanical meaning of the paraspinal musculature on the IVD completely. Also, potentially diffusion tensor imaging for predicting muscle strength (29) or muscle tissue sampling, including histological examination, might be helpful to prove this hypothesis. These MRI techniques were unavailable at our institution, and muscle tissue sampling was an invasive technique and was also unavailable due to the retrospective character of the study.

To conclude, this study aimed to clarify the possible impact of the cervical paraspinal musculature of FBs on cervical IVDH development. This would be important to evaluate the clinical role, especially in a predisposed breed, to develop preventive therapeutic optimization and strategies for IVDH patients, such as physiotherapeutic interventions. Although some differences in the paraspinal musculature between FBs, with and without IVDH, were detected in the height ratio, most results were not statistically significant between these two groups. Hence, there is a suspicion that other factors play a more important role in developing IVDH in this breed. Statistically significant differences were determined in height and area ratios, angulation, and the relation of moments between FBs and other breeds. Especially at the level of C3/C4, which is the most affected site for cervical FB IVDH (3), steeper angulation of the IVD combined with less prominent dorsal paraspinal musculature was observed. This induces the hypothesis that the paraspinal musculature might be an additional anatomical and biomechanical influencing factor on preferential IVDH sites in the cervical spine. Further studies are required to fully explain the pathophysiological effect of the cervical paraspinal musculature on IVDH in these patients.

## Data Availability Statement

The original contributions presented in the study are included in the article/Supplementary Materials, further inquiries can be directed to the corresponding author.

## Ethics Statement

Ethical review and approval were not required for this study because all CT-scans of the patients were performed for reasons unrelated to this study. Written informed owner consent and consent for participation was not obtained according to the retrospective character of the study.

## Author Contributions

JH: study design, measurements, generation of the manuscript, and statistical analysis. FF: study design and correction of the manuscript. PD: correction of the manuscript. CF and KH: generation and correction of the manuscript. SR: provision of computed tomographic images and correction of the manuscript. All authors contributed to the article and approved the submitted version.

## Conflict of Interest

The authors declare that the research was conducted in the absence of any commercial or financial relationships that could be construed as a potential conflict of interest.

## Publisher's Note

All claims expressed in this article are solely those of the authors and do not necessarily represent those of their affiliated organizations, or those of the publisher, the editors and the reviewers. Any product that may be evaluated in this article, or claim that may be made by its manufacturer, is not guaranteed or endorsed by the publisher.
