# Supplementary material for: Comparison of Dorsal-to-Ventral Ratios of the Cervical Paraspinal Musculature in French Bulldogs With and Without Cervical Intervertebral Disk Disease and Two Other Breeds Based on CT Scan Measurements
Source: Front Vet Sci. 2021 Nov 22;8:705632. doi: 10.3389/fvets.2021.705632 (PMC8645773; doi:10.3389/fvets.2021.705632)
Supplement: Supplementary file 1 [file Data_Sheet_1.DOCX]

Supplementary Material

# Supplementary Data

**Supplementary Table 3.** Supplementary data of the study by Hartmann et al. (2020) of Height ratio, Angles, Area ratio, and Ratio of moments of Labrador Retrievers (L) and Dachshunds (D).
